# Protein intake and clinical outcomes in critically ill patients: A dose–response and pairwise meta-analysis of randomized controlled trials

**DOI:** 10.34172/hpp.025.43894

**Published:** 2025-07-15

**Authors:** Mohaddeseh Badpeyma, Faezeh Ghalichi, Roghayeh Molani-Gol, Hamed Valizadeh, Yousef Javadzadeh, Ahmadreza Rasouli, Mohammad Alizadeh, Sorayya Kheirouri

**Affiliations:** ^1^Department of Clinical Nutrition, Faculty of Nutrition and Food Sciences, Tabriz University of Medical Sciences, Tabriz, Iran; ^2^Student Research Committee, Tabriz University of Medical Sciences, Tabriz, Iran; ^3^Department of Nutrition and Food Sciences, Maragheh University of Medical Sciences, Maragheh, Iran; ^4^Tuberculosis and Lung Diseases Research Center, Tabriz University of Medical Sciences, Tabriz, Iran; ^5^Biotechnology Research Center and Faculty of Pharmacy, Tabriz University of Medical Sciences, Tabriz, Iran

**Keywords:** Critical illness, Dietary proteins, Intensive care units, Meta-analysis, Mortality, Muscle atrophy, Nutritional support, Randomized controlled trials

## Abstract

**Background:**

The optimal protein intake for critically ill patients remains uncertain. This systematic review and dose–response meta-analysis aimed to evaluate the effect of high-protein nutritional support on clinical outcomes in intensive care unit (ICU) patients.

**Methods:**

Randomized controlled trials (RCTs) comparing high- versus low-protein nutrition in critically ill adults with similar energy intake were identified through PubMed, Web of Science, and Scopus (up to June 2023). A random-effects model was used to pool risk ratios (RRs) and mean differences (MDs) with 95% confidence intervals (CIs). Linear and non-linear trends were assessed using the one-stage cubic spline regression model.

**Results:**

Twenty-three RCTs were included. The summary RR was 0.83 (95% CI: 0.64–1.08; I^2^=63.6%; n=17) for mortality and 1.05 (95% CI: 0.88–1.25; I^2^=0%; n=7) for infections. The summary MD was -0.23% (-0.76 to 0.29, I^2^=5.6%, n=14) for mechanical ventilation days, -0.40 (-1.11 to 0.32, I^2^=0%, n=17) for ICU days, 0.73 (-1.11 to 2.58, I^2^=6%, n=10) for hospital days, and -3.44 (-4.99 to -1.90; I^2^=16.4%; n=5) for muscle atrophy. There was no evidence of linear or nonlinear trends.

**Conclusion:**

Although higher protein intake had no significant effect on mortality or length of stay, it was associated with reduced muscle wasting. This suggests a potential role in preserving lean mass and supporting long-term functional recovery.

**Systematic Review Registration:**

PROSPERO CRD42024480303.

## Introduction

 Critical illness is characterized as a severe catabolic process leading to increased mortality and the development of both infectious and noninfectious complications.^1^ Nutrition has an efficient effect in declining and controlling morbidity.^2-4^ Early initiation of nutrition therapy and sufficient energy and nutrient intake significantly affect short-term and long-term endpoints. ^1^ During intensive care unit (ICU) stay, malnutrition and muscle wasting are common. This may be due to catabolic hormones, imbalance in intake and requirements, and physical inactivity.^5,6^ In critical illness, fat recruitment, breakdown of protein, and hyperglycemia cause metabolic stress.^7^

 Protein is the most important macronutrient as it potentiates healing and immune function and helps patients maintain lean body mass.^8^ Thus, high protein intake after the commencement of critical conditions may efficiently decline endogenous proteolysis, which results in the preservation of muscle mass. Despite such evidence and interest in the beneficial health effects of protein intake, dietary guidelines did not provide consistent recommendations. For example, ESPEN and ASPEN guidelines recommend a daily intake of 1.3 g/kg body weight/day^9^ and 1.2–2.0 g/kg/day, respectively.^10^ In accordance with US-American guidelines, higher protein doses (2.0–2.5 g/kg BW/d) are considered safe and may be beneficial for certain subgroups of patients in the ICU.^11-14^ However, observational data indicates that many patients do not meet these recommended protein targets. According to multiple studies, patients receiving standard critical care management only receive an average of 0.6–0.8 g/kg/d, most likely as a result of feeding interruptions, intolerance, and limited access to higher protein formulas.^15-18^

 Previous systematic reviews and meta-analyses regarding the optimal protein dose in critical illnesses have yielded conflicting results and created further confusion. For example, Zou et al reported that higher protein delivery significantly reduces muscle loss (3.4% per week) and may be associated with a shorter ICU length of stay; however, there was no effect on mortality, infectious complications, or hospital length of stay. Davies et al. included 14 randomized controlled trial (RCT) of artificial nutritional support with 3238 patients and found no effect of lower (0.67 ± 0.38 g/kg/day) versus higher protein (1.02 ± 0.42 g/kg/day) provision on mortality.^9^ Higher protein intake also did not significantly affect ICU endpoints in two other meta-analyses.^19^ However, a recent large-scale RCT found that high protein intake did not improve the time-to-discharge-alive from the hospital and may have even worsened outcomes for patients with acute kidney injury and high organ failure scores, following the publication of several meta-analyses on the topic.^20^ Combining new data from this large RCT with previous ones is important to provide the most up-to-date evidence. While the dose of protein intake is of central importance, previous meta-analyses focused only on traditional pairwise comparisons between intervention and control groups and ignored the prescribed amount of protein. Since different amounts of protein may have different effects, clarifying the shape and strength of the dose-response association throughout the entire range of protein is important from a clinical point of view. Therefore, the current systematic review and meta-analysis aimed to assess the dose-response relationship between prescribed protein and clinical endpoints in ICU patients.

## Material and Methods

 For the purpose of reporting this systematic review, we adhered to the PRISMA (Preferred Reporting Items for Systematic Reviews and Meta-Analyses) guideline.^21^ We registered the protocol on the International Prospective Register of Systematic Reviews (PROSPERO CRD42024480303).

###  Search strategy

 We searched the PubMed, Web of Science, and Scopusdatabases until June 23, 2023. Two reviewers developed and performed the literature search (M.B. and R.M.), and two reviewers (M.B. and R.M.) screened the publications. Details of the search terms are provided in Supplementary file 1, Table S1. Additionally, we manually searched past meta-analyses and the reference lists of the included research to make sure we didn’t overlook any relevant studies. Our systematic search was limited to English-language studies.

###  Inclusion and exclusion criteria

 The inclusion criteria for studies were as follows: 1) Randomised controlled trials including adults aged 18 years and over admitted to an intensive care unit; 2) Studies that examined the impact of high-dose protein nutritional support enteral nutrition (EN), parenteral nutrition (PN), or a combination of both (EN and PN) compared to the usual doses of protein in ICU patients; 3) Studies that provided risk estimates, means, and standard deviations (SDs) for the outcomes of interest or provided enough data to calculate these values. Observational and ecological research, reviews, letters, comments, and meta-analyses were excluded. Studies that reported effect estimates for high protein formulas in combination with other interventions were also not included. Additionally, unpublished studies and gray literature were excluded from the meta-analysis.

###  Data extraction

 Two researchers (M.B. and R.M.) independently reviewed and extracted data from the included studies. A third reviewer (M.A.) was consulted to settle any disagreements. The following data were extracted from each study: study characteristics (first author’s name, year of publication, and study location), participant characteristics (mean age or age range, gender, the health condition of the participants, and sample size), intervention (type, dose, and duration of intervention), and outcome data.

###  Outcomes 

 At the longest follow-up, overall mortality was the primary outcome. Mortality was selected as the primary outcome because it is not prone to ascertainment bias. Data were also extracted for the following secondary outcomes: duration of mechanical ventilation, length of stay in the ICU and hospital, infectious complications, and muscle atrophy.

###  Quality assessment of studies

 Using the Cochrane Risk of Bias Tool,^22^ two investigators (F.G. and A.R.) independently assessed the risk of bias in the trials included in the study. The evaluation considered several methodological domains, including random sequence generation, allocation concealment, blinding of participants and personnel, blinding of outcome assessment, incomplete outcome data, selective reporting, and other potential threats to validity. Each domain was categorized as having a low, unclear, or high risk of bias. An overall quality score was given to the trials based on bias domains: Low risk of bias ( ≤ 1 items was unknown and none were high), Some concern ( ≤ 2 items were unclear or at least one high), and high risk of bias ( ≥ 2 items were high).

###  Statistical analysis

 The mean and standard deviation were used to record continuous data. In cases where median data were reported, they were converted to mean data along with corresponding variances using established methods.^23,24^ For binary outcomes, we extracted the number of patients who had each outcome and divided it by the total number of patients in that group. Continuous variables were reported as mean differences (MDs) or standardized mean differences (SMDs) with a 95% confidence interval (CI), while dichotomous variables were presented as pooled risk ratios (RRs) with a 95% CI. All analyses were conducted using an inverse variance random-effects model. Heterogeneity was assessed using the Cochran Q statistic and quantified using the I^2^ statistic. A significance level of *P* < 0.10 was used to determine heterogeneity, with an I^2^ value greater than 50% indicating substantial heterogeneity.^25^ We conducted pre-specified subgroup analyses based on study location (western vs non-western countries) and type of intervention (EN vs. PN) to identify potential sources of heterogeneity. We also conducted a sensitivity analysis, where each study was excluded one by one to assess the impact of that study on the overall effect estimate. To examine small study effects, such as publication bias, we visually inspected funnel plots. Using Egger’s regression asymmetry test, funnel plot asymmetry was also formally statistically evaluated. Moreover, we performed a linear dose-response analysis using a one-stage weighted mixed effects meta-analysis. Non-linear dose-response relationships were evaluated using restricted cubic splines with 3 knots based on Harrell’s recommended percentiles (10%, 50%, and 90%) of the distribution.^26^ All statistical analyses were carried out using Stata software, version 17.0 (Stata Corp LP, College Station, TX, USA). A p-value less than 0.05 was considered statistically significant for all tests.

## Results

###  Literature search and study characteristics

**Figure 1:**
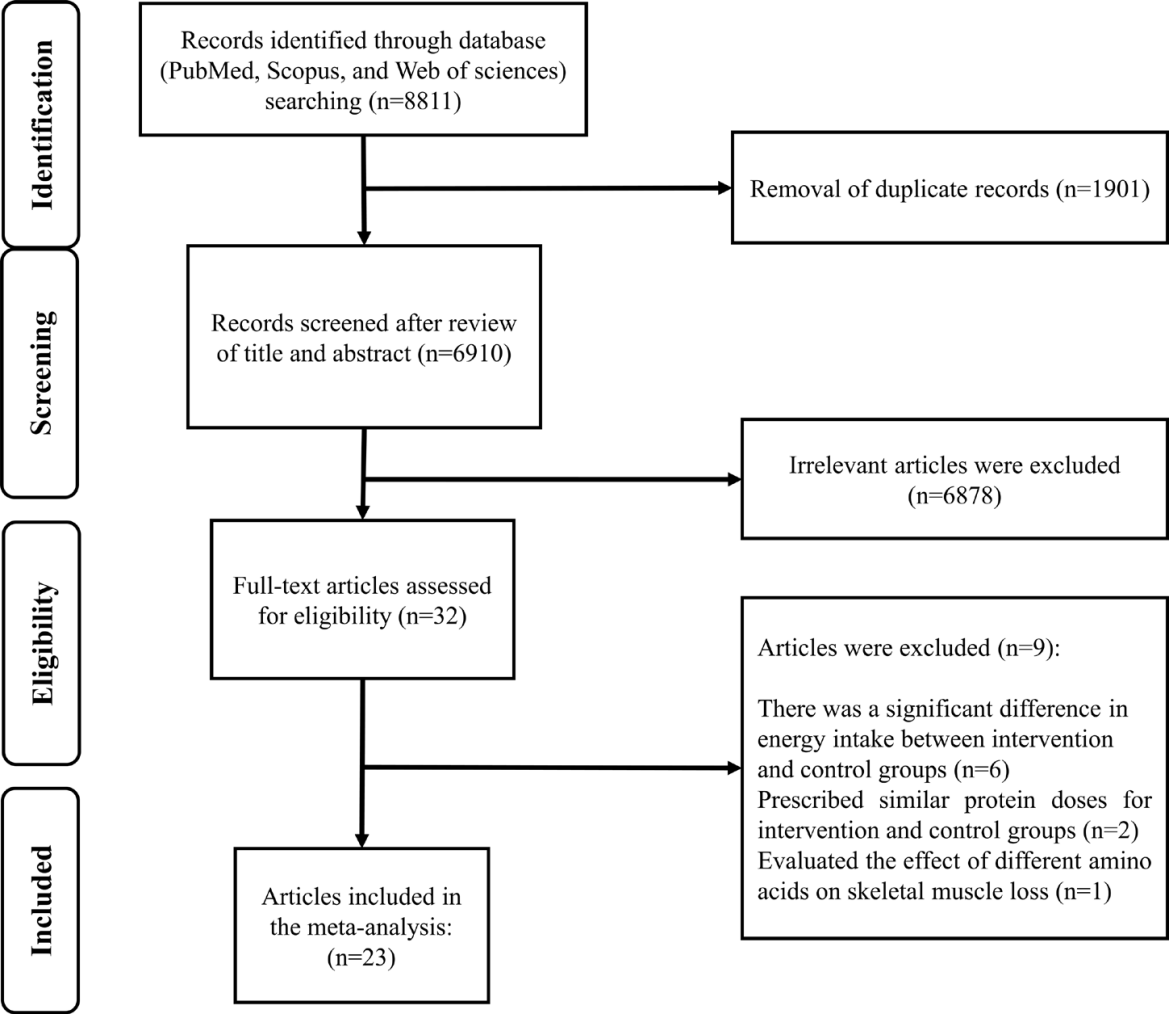
Flow diagram of the literature search and study selection process

 Out of the 32 publications that were thoroughly reviewed, 9 studies were excluded based on the eligibility criteria. We excluded studies that showed a significant difference in energy intake between the intervention and control groups,^27-32^ studies that prescribed similar protein doses for the groups being studied,^33,34^ and studies that focused on the effect of different amino acids on skeletal muscle loss.^35^ Ultimately, 23 studies were included in the current systematic review and meta-analysis (Figure 1). The general characteristics of these included trials are provided in Supplementary file 1, Table S2. The publication years of the included studies ranged from 2007 to 2023, and all of them followed a parallel design. The trials involved a varying number of participants, ranging from 14 to 1301, with ages ranging from 19 to 101 years. In total, 3324 participants were included in the meta-analysis. Among the included studies, 16 were carried out in Western and 7 in non-Western countries. All trial comparisons were conducted on both sexes. The intervention duration varied from 3 to 28 weeks. Units of protein intake varied in different studies (g/kg/day, %E, or g/day). The amount of prescribed protein in the control group ranged from 0.8 to 1.55 g/kg/day (14% to 22% E), and in the intervention group ranged from 1.2 to 2.4 g/kg/day (16% to 32% E). Using the Cochrane quality assessment tool, nine studies have a high risk of bias, eleven have an unclear risk of bias, and three have a low risk of bias (Supplementary file 1, Table S3).

###  Findings from meta-analysis 

####  Mortality

**Figure 2:**
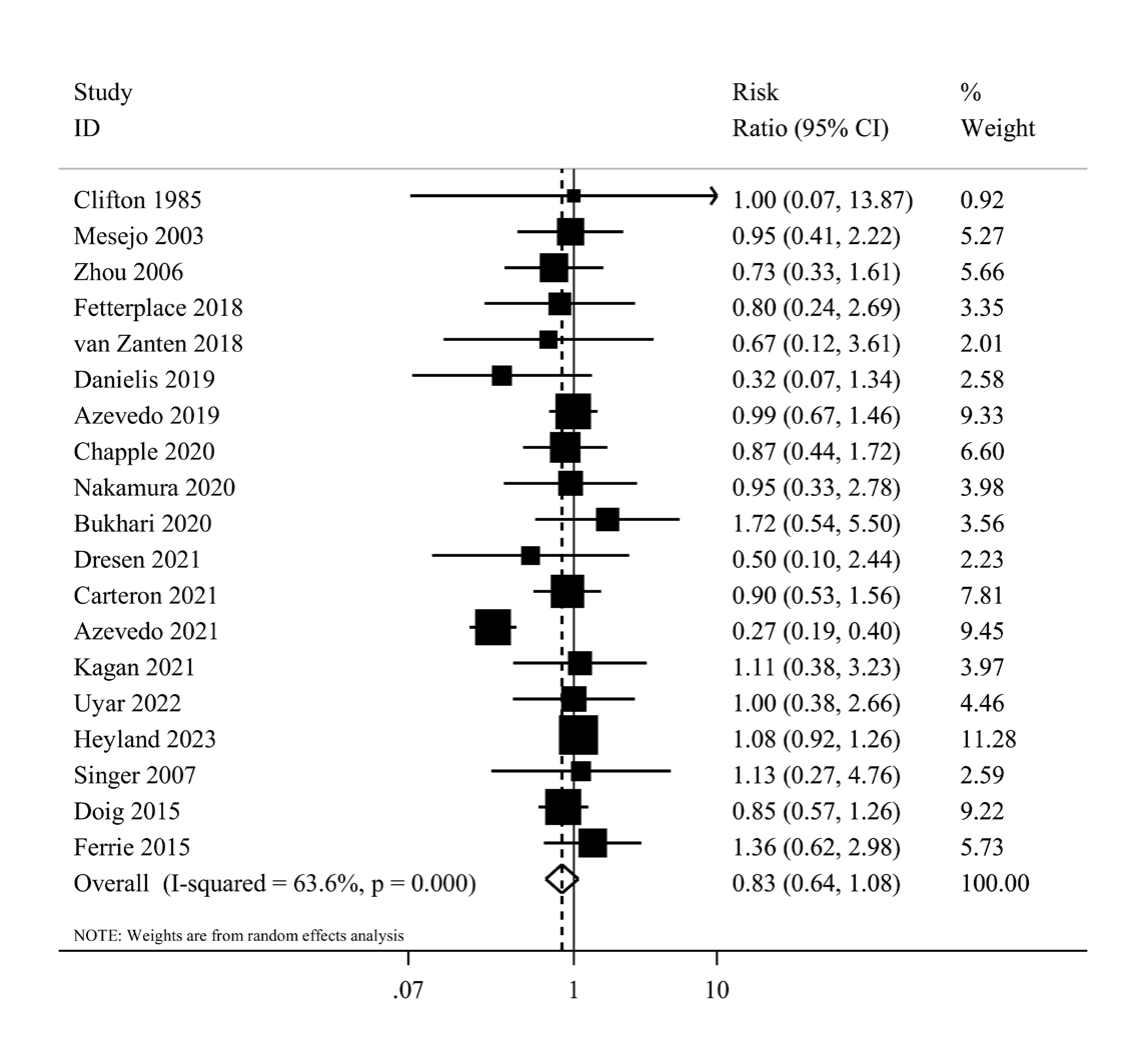
Forest plot of the overall mortality for high protein delivery compared with low protein delivery.Horizontal lines represent 95% of CIs Diamonds represent the pooled estimates from the random-effects analysis.CI: confidence interval

**Figure 3:**
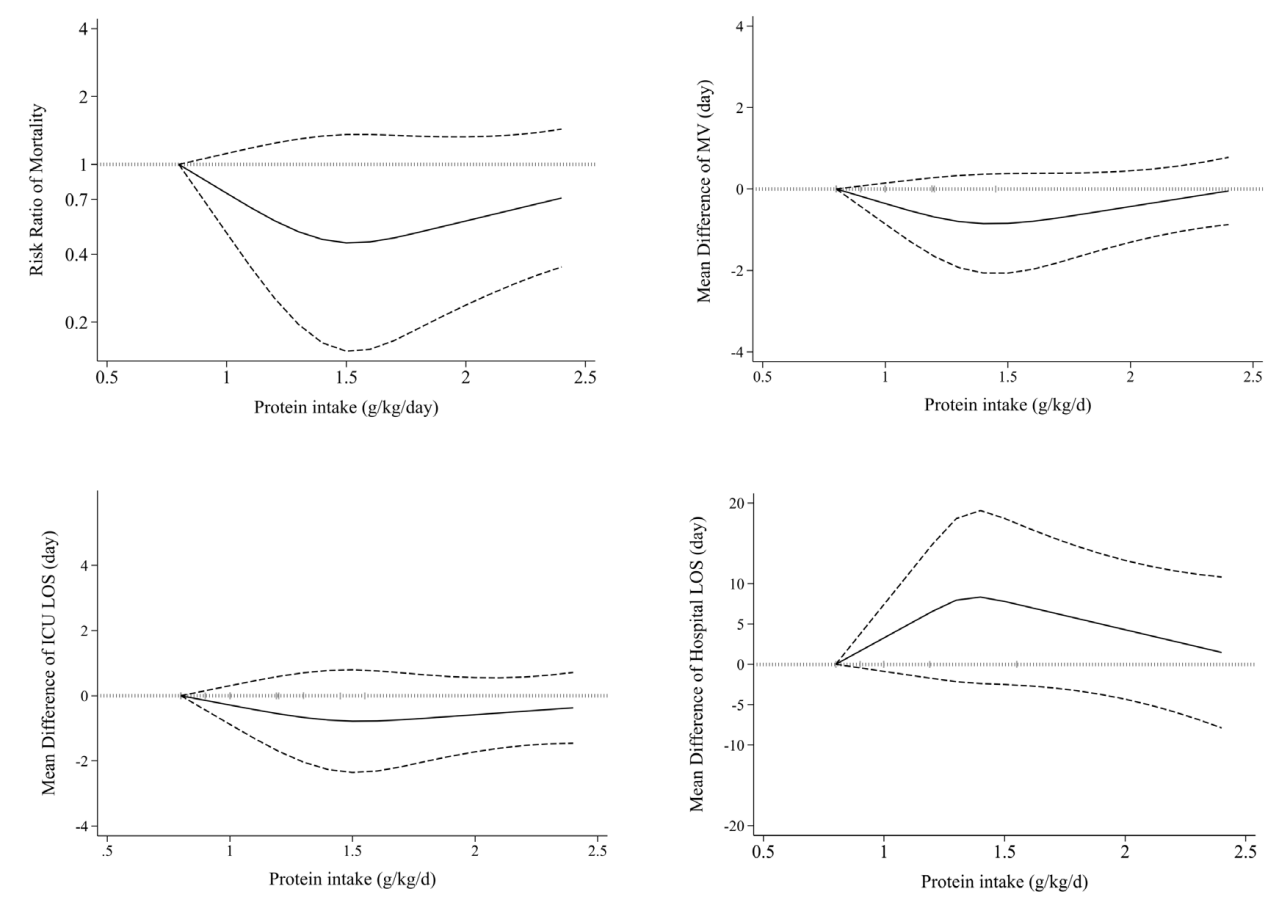
Non-linear dose-response effects of high protein intake on ICU outcomes. ICU: Intensive Care Unit, MV: Mechanical Ventilation, LOS: length of stay

 Nineteen studies^1,7,8,36-38,40,41,43,44,46,48-55^ examined the effects of high protein intake on mortality in comparison with low protein intake. These studies included a total of 3056 patients. Combining data from these studies demonstrated no significant effect of higher protein intake on mortality (RR = 0.83, 95% CI: 0.64 to 1.08, I^2^ = 63.6%, *P* < 0.001) (Figure 2). The summary RR per 0.2 g/kg/day was 0.91 (0.81 to 1.02; I^2^ = 80.6%, *P* < 0.001) (Supplementary file 1, Figure S1). There was no evidence of departure from linearity (*P*_nonlinearity_ = 0.15) (Figure 3).

 Subgroup analyses were performed to test the robustness of the findings and evaluate the possible sources of heterogeneity. Supplementary file 1, Table S4 shows the results for different subgroups. The summary RR was 0.79 (95% CI: 0.58 to 1.09, I^2^ = 68.9%, *P* < 0.001) for enteral and 0.94 (95% CI: 0.67 to 1.33, I^2^ = 0, *P* = 0.56) for parenteral feeding. Moreover, there was no statistically significant difference in the association between Western (RR = 0.77, 95% CI: 0.55 to 1.08, I^2^ = 74.8%, *P* < 0.001) and non-western countries (RR = 1.00, 95% CI: 0.65 to 1.53, I^2^ = 0%, *P* = 0.91) in the stratified analyses. In sensitivity analysis, excluding one study at a time from the analysis did not appreciably alter the summary RR. The results of the Egger’s test did not provide any evidence of small study effects (*P* = 0.47), and upon visual inspection, no asymmetry was observed in the funnel plot.

####  Infectious complications

**Figure 4:**
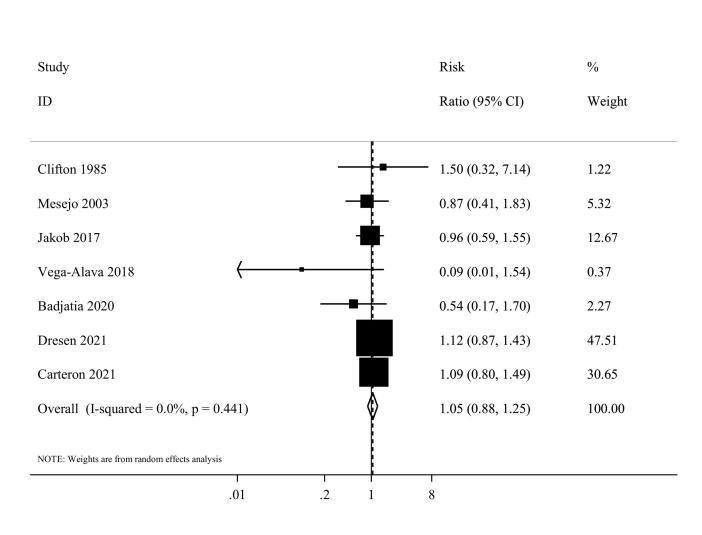
Forest plot of the infectious complications for high protein delivery compared with low protein delivery.Horizontal lines represent 95% of CIs Diamonds represent the pooled estimates from the random-effects analysis.CI: confidence interval

 Information on protein intake and infectious complications was available in seven studies,^36,37,42,45,47,51,52^ with a total of 462 critically ill patients. The summary RR for infectious complications was 1.05 (95% CI: 0.88 to 1.25, I^2^ = 0%, P = 0.44) (Figure 4). A dose-response analysis was not performed due to the limited number of studies.

 In the subgroup analyses, the summary RR was 1.06 (95% CI: 0.89 to 1.26, I^2^ = 0%, *P* = 0.80) for Western countries and 0.09 (95% CI, 0.01 to 1.54) for non-western countries (Supplementary file 1, Table S4). In sensitivity analyses, excluding each study individually did not significantly alter the estimated summary. Egger’s test also indicated no evidence of small study effects or publication bias (*P* = 0.06). Furthermore, the visual inspection of the funnel plot did not reveal any obvious publication bias.

####  Duration of mechanical ventilation

**Figure 5:**
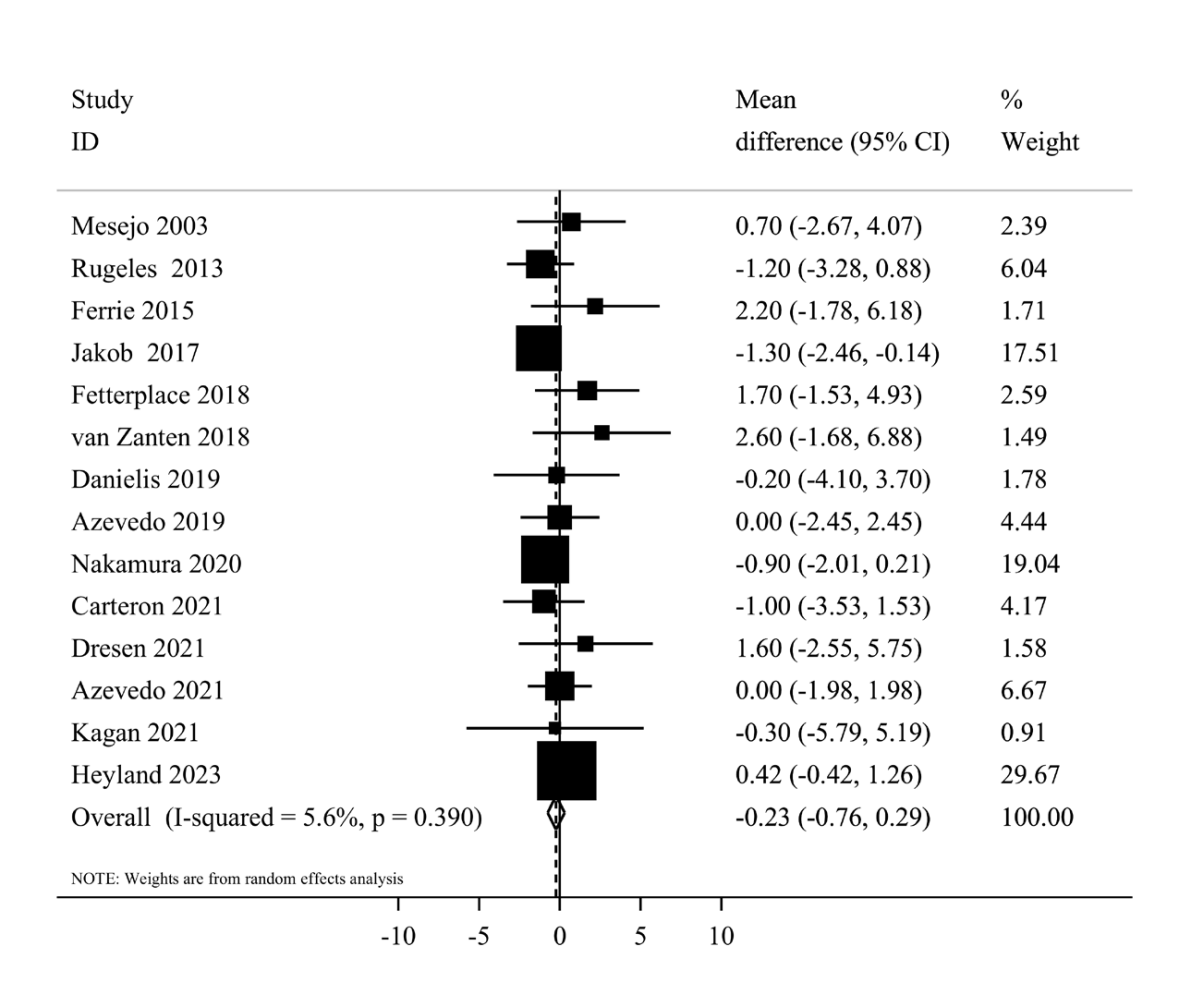
Forest plot of the duration of mechanical ventilation for high protein delivery compared with low protein delivery.Horizontal lines represent 95% of CIs Diamonds represent the pooled estimates from the random-effects analysis.CI: confidence interval

 Fourteen eligible clinical trials,^7,20,37,39,41-44,46,50-54^ including 2479 ICU patients, investigated the mechanical ventilation duration among higher and lower protein groups. Combining data from these studies indicated that the duration of mechanical ventilation was not different between the two groups (MD = -0.23, 95% CI: -0.76 to 0.29, I^2^ = 5.6%, *P* = 0.39) (Figure 5). The dose-response meta-analysis showed no significant difference per 0.2 g/kg/day increase in protein intake (MD = 0.01 day; 95%CI: -0.10 to 0.11, I^2^ = 0%, *P* = 0.47) (Supplementary file 1, Figure S2). Moreover, the result of the non-linearity test was not significant (*P*_non-linearity_ = 0.14) (Figure 3).

 In terms of subgroup analyses, the summary MD was similar among studies that used enteral (MD = -0.27, 95% CI: -0.78 to 0.24, I^2^ = 2.6%, *P* = 0.42) and parenteral (MD = 2.20, 95% CI: -1.78 to 6.18) nutrition therapy. Moreover, the summary MD was -0.07 (95% CI: -0.69 to 0.54, I^2^ = 8.3%, *P* = 0.36) for Western countries and -0.88 (95% CI: -1.96 to 0.21, I^2^ = 0.0%,* P* = 0.83) for non-western countries (Supplementary file 1, Table S4). Sensitivity analysis, where each study was excluded one at a time, did not substantially change the pooled effect estimate in terms of significance, magnitude, or direction. Egger’s test did not detect any publication bias (*P* = 0.25).

####  Length of ICU stay

**Figure 6:**
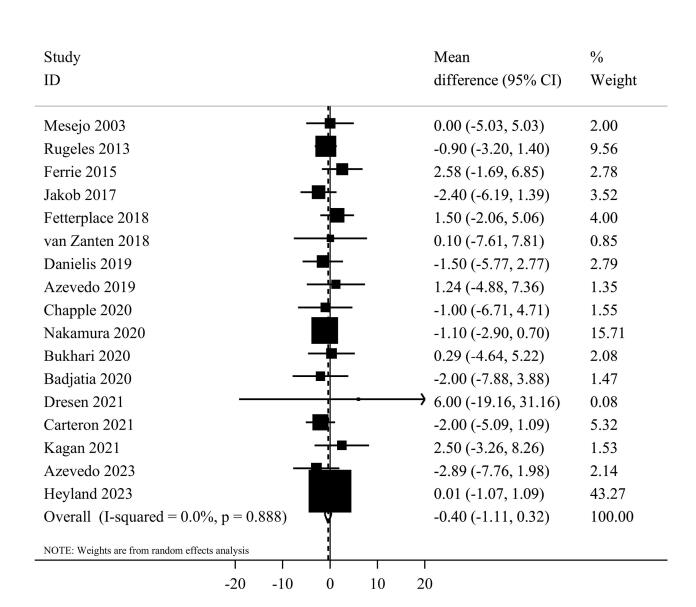
Forest plot of the length of ICU stay for high protein delivery compared with low protein delivery.Horizontal lines represent 95% of CIs Diamonds represent the pooled estimates from the random-effects analysis.CI: confidence interval

 Seventeen studies^7,20,37,39,41-44,46-54^ comprising 2644 critically ill patients provided data for the length of ICU stay. Summarizing data from these studies showed no significant differences between studied groups (MD = -0.40, 95% CI: -1.11 to 0.32, I^2^ = 0%, *P* = 0.88) (Figure 6). In the linear trend estimation, there was no significant difference per 0.2 g/kg/day increase in protein intake (MD = -0.04 day; 95%CI: -0.18 to 0.09, I^2^ = 0%, *P* = 0.77) (Supplementary file 1, Figure S3). Moreover, there was no evidence of a non-linear dose-response association (*P*_non-linearity_ = 0.40) (Figure 3).

 With regard to sub-group analyses, the summary effect estimate was -0.48 (95% CI: -1.20 to 0.24, I^2^ = 0.0%, P = 0.93) for enteral and 2.58 (95% CI: -1.69 to 6.85) for parenteral nutrition therapy. Moreover, the summary results were similar among western (MD = -0.21, 95% CI: -1.03 to 0.61, I^2^ = 0.0%, *P* = 0.87) and non-western (MD = -0.95, 95% CI: -2.39 to 0.48, I^2^ = 0.0%, *P* = 0.55) countries (Supplementary file 1, Table S4). In the sensitivity analysis, the removal of each trial did not change the significance, magnitude, or direction of the estimated effect size. According to Egger’s tests, there was no significant publication bias (*P* = 0.92).

####  Length of hospital stay

**Figure 7:**
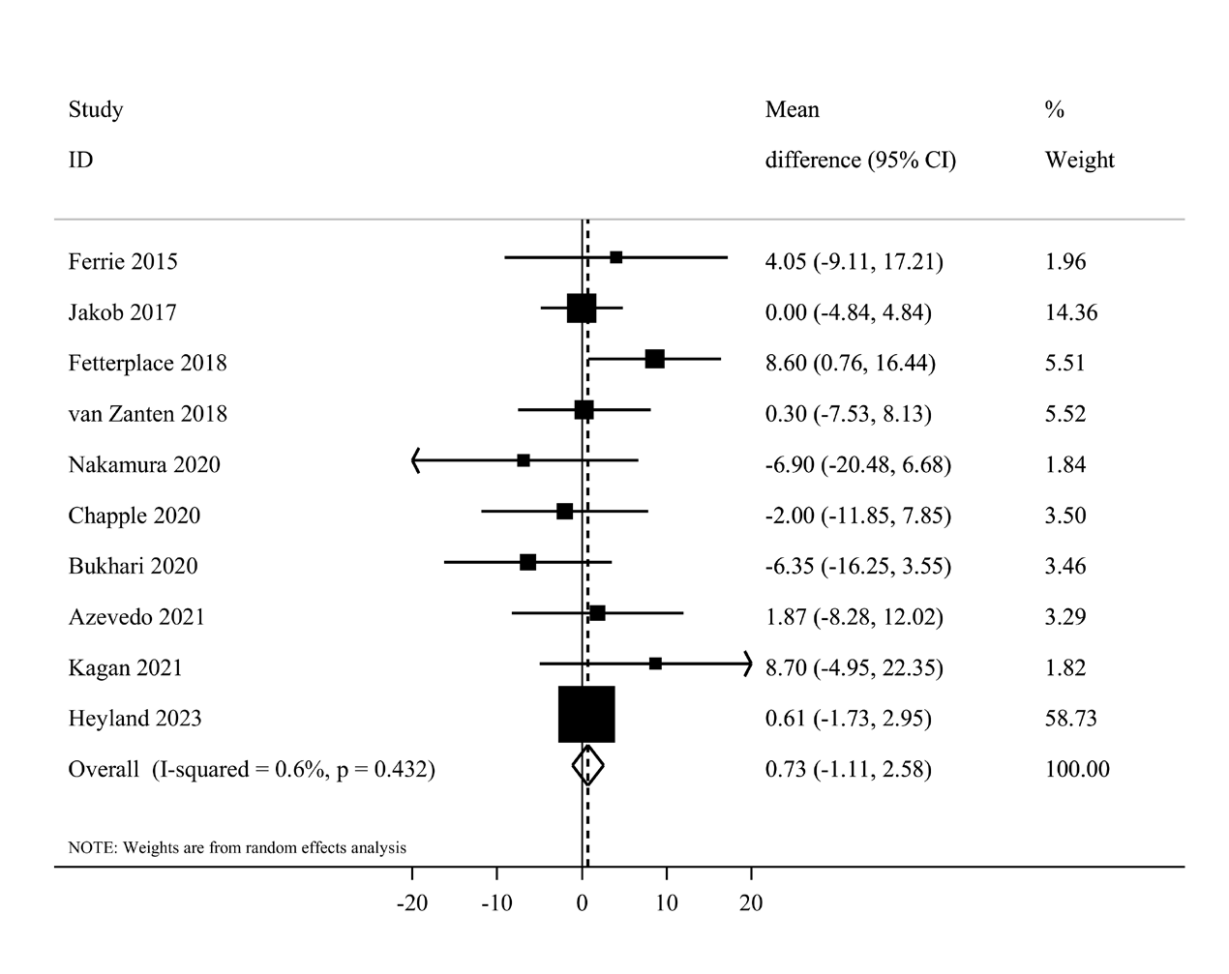
Forest plot of the length of hospital stay for high protein delivery compared with low protein delivery.Horizontal lines represent 95% of CIs Diamonds represent the pooled estimates from the random-effects analysis.CI: confidence interval

 Ten eligible^20,41-44,48-50,54,53^ clinical trials, including 2092 patients, investigated the effect of higher protein delivery on the length of hospital stay compared to low protein intake. Combining findings from these studies indicated no significant difference in the length of hospital stay between the two groups (MD = 0.73, 95% CI -1.11 to 2.58, I^2^ = 0.6%, *P* = 0.432) (Figure 7). The results of the linear dose-response analysis demonstrated that every 0.2 g/kg/day increase in protein intake was not associated with length of hospital stay (MD = 0.16 day; 95% CI: -1.28 to 1.59, I^2^ = 35.6%, *P* = 0.17) (Supplementary file 1, Figure S4). Moreover, a non-linear association was not evident between protein delivery and length of hospital stay (*P*_non-linearity_ = 0.11) (Figure 3).

 Based on the subgroup analyses, the summary MD was 0.68 (95% CI: -1.52 to 2.89, I^2^ = 9.2%, P = 0.35) for enteral and 4.05 (95% CI -9.11 to 17.21) for parenteral feeding. Moreover, a non-significant difference was reached among western (MD = 0.98, 95% CI, -0.90 to 2.86, I^2^ = 0%, *P* = 0.60) and non-western (MD = -2.11, 95% CI: -11.55 to 7.34, I^2^ = 43.9%, *P* = 0.16) countries (Supplementary file 1, Table S4). The sensitivity analysis confirmed that the overall pooled effect remained unchanged after removing each study. Based on Egger’s test (P = 0.88), there was no evidence of publication bias.

####  Muscle atrophy

**Figure 8:**
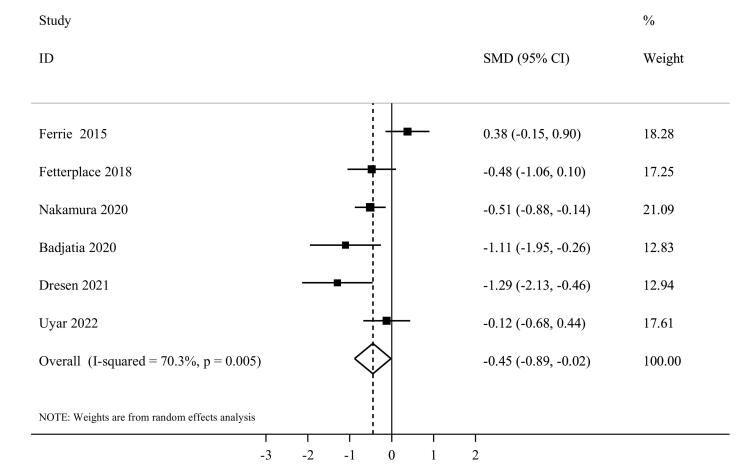
Forest plot of the muscle atrophy for high protein delivery compared with low protein delivery.Horizontal lines represent 95% of CIs Diamonds represent the pooled estimates from the random-effects analysis.CI: confidence interval

 Six studies^1,41,43,47,50,52^ reported sufficient data for muscle atrophy and included a total of 273 participants. The summary effect estimates of their findings using the random effects model indicated that higher protein delivery significantly attenuated muscle loss in comparison with lower protein delivery (SMD = -0.45, 95% CI, -0.89 to -0.02, I^2^ = 70.3%, *P* = 0.005) (Figure 8). In terms of sub-group analysis, a significant effect was seen among studies that administered enteral feeding (SMD = -0.60, 95% CI: -0.95 to -0.24, I^2^ = 43.3%, *P* = 0.13) (Supplementary file 1, Table S4). After excluding each individual study using sensitivity analysis, no significant difference in the summary effect estimate was found. Based on Egger’s test (*P* = 0.88), we found no substantial publication bias.

## Discussion

 The present systematic review and meta-analysis synthesized the findings of 23 RCTs, including 3324 critically ill patients. This study demonstrated that prescribing nutritional support with high doses of protein compared with usual doses of protein in critically ill patients did not improve mortality, infectious complications, duration of mechanical ventilation, ICU, and hospital length of stay. Observational studies have revealed a positive association between high protein intake and improved muscle mass, clinical outcomes, and physical performance in critically ill participants. Nevertheless, such studies are not able to discern whether higher protein intake plays a causal role in preventing mortality and other endpoints in ICU patients. RCTs are able to overcome the limitations of observational studies, and consequently, their findings provide complementary evidence. The current meta-analysis, which includes four additional studies and a 90% increase in sample size compared to previous ones, is among the first comprehensive meta-analyses to investigate the effect of high-protein nutritional support on clinical outcomes in critically ill patients.

 In terms of overall mortality as a primary outcome, prescribing high doses of protein did not reduce the risk of mortality. The last meta-analysis,^56^ which was published in 2021, reported that higher vs. lower protein did not significantly affect overall mortality. This meta-analysis with similar inclusion criteria included 19 studies, 15 of which (1492 participants) reported data on mortality. As compared with this meta-analysis, four studies were additionally included in our analysis.^1,20,54,55^ Therefore, our study, with a total of 3056 participants and 845 mortality cases, provided more accurate and comprehensive estimates of the protein effects. In another meta-analysis, higher protein delivery to ICU patients did not reduce the risk of mortality. However, this review included only six studies and restricted their analyses to studies that provided predominately enteral nutrition.^19^ Moreover, they included studies showing significant differences in caloric intake between the high- and low-protein groups.^9,19^ The difference in energy intake between the control and intervention groups can affect the independent effect of protein intake on the investigated outcomes.

 In terms of secondary outcomes, a non-significant effect was obtained for infectious complications, duration of mechanical ventilation, ICU, and hospital length of stay. Previous meta-analyses have generally reported findings similar to ours. For instance, higher protein doses in critically ill patients did not affect the length of ICU stay, mechanical ventilation, and incidence of infections in a meta-analysis of Lee et al.’s study.^56^ According to Fetterplace et al’s systematic review and meta-analysis, mean enteral protein intake in a limited dataset of critically ill patients that was approximately at the lower end of the range recommended by international guidelines did not appear to reduce mortality or shorten the length of acute admission when compared to usual treatment.^19^ Overall, evidence from RCTs consistently shows that in critically ill patients, nutritional support with high doses of protein compared with standard doses of protein could not affect the clinical outcome. However, higher protein compared to lower protein delivery reduced muscle atrophy in this study. Several mechanisms have been mentioned: Critical illness leads to systemic inflammation, increasing levels of C-reactive protein (CRP), interleukin-6 (IL-6), and tumor necrosis factor-alpha (TNF-α), all of which contribute to muscle degradation. Studies indicated that inflammatory cytokines accelerate protein breakdown in muscle mass. Protein supplementation helps counteract this by maintaining anabolic signaling.^57-59^ Oxidative stress caused by elevated reactive oxygen species (ROS) and nitric oxide further exacerbates muscle breakdown. High-protein diets can help neutralize oxidative stress and inhibit catabolic pathways like nuclear factor kappa-B (NF-κB), which is known to accelerate muscle deterioration.^60^ Additionally, high-protein intake, particularly branched-chain amino acids (BCAAs) like leucine, activates the mTOR (mammalian rapamycin target) pathway, promoting muscle protein synthesis.^58,61^ Callahan et al reported that leucine plays a key role in muscle anabolism by stimulating muscle protein synthesis (MPS), which is crucial in preventing muscle wasting in critically ill patients.^62^ In critically ill patients, muscle loss is caused mainly by muscle fiber atrophy and apoptosis. Puthucheary et al demonstrated that ICU patients experience muscle wasting at a rate of 1%–2% per day,^63^ and another study claimed that protein supplementation helps slow down apoptosis and atrophy.^64^ In addition, protein intake is essential for wound healing, immune function, and rehabilitation. During immobilization, protein supplementation helps maintain muscle integrity and improves post-illness recovery.^53,65^ Therefore, the provision of exogenous amino acid by enhancing the central and peripheral synthesis of protein, optimizing response to inflammation, modifying the extensive muscle protein loss in the short term, and minimizing muscle atrophy in the long term could improve critical illness.^66-68^

 Previous meta-analyses have attempted to investigate the effectiveness of high-dose protein delivery on clinical endpoints among critical illness patients, but they have been unable to fully characterize it because they have only performed traditional pairwise meta-analyses, and none of them examined the dose-dependent effect in their analyses.^9,43,19^ In this study, the dose-response relationships between prescribed protein and outcomes of interest over the entire exposure range were investigated. Therefore, our meta-analysis, which assessed the dose-response associations, provided the most updated knowledge about the effect of higher protein delivery in ICU patients. The present study did not reveal any linear or non-linear dose-deepening effect between protein intake and investigated outcomes assessed. Nevertheless, these findings should be interpreted with caution because most included trials had a dose of around 1.3 g/kg/d, and the slope of the regression line was essentially determined by a few distinctive doses within a narrow range, which may not be sufficient to delineate the underlying dose-response associations.

 Heyland et al^20^ indicated that receiving high doses of protein was not efficient in improving clinical outcomes among patients in ICUs. This study, which recruited 1301 patients from 85 ICUs in 16 countries, comprised more than 40% of the sample size of the current study. Because of the large sample size of the Heyland et al^20^ study, its effect estimates gained a higher weight in the analysis compared with other ones. To address this issue, a sensitivity analysis was performed, in which we excluded the Heyland et al^20^ study to examine the effect of that study on all the findings. The results showed that removing this study from the analysis did not appreciably alter the summary effect estimates (data not shown).

 The current meta-analysis has several important strengths. Firstly, different types of analyses were used to assess the effect of high-protein intakeon different clinical outcomes, including pairwise, linear, and non-linear dose-response meta-analyses. Secondly, in comparison to prior meta-analysis studies, our study consisted of the latest studies and, therefore, updated previous results. Thirdly, the inclusion of studies with an RCT design allowed us to draw causal conclusions with minimal bias. Fourthly, our findings were robust to sensitivity and subgroup analyses. Finally, patients with diverse diseases who were admitted to multiple ICU settings were included, which increases the generalizability of our findings. The findings of the current meta-analysis should be interpreted considering the following limitations: This study might not be powerful enough to assess the dose-dependent effect of protein due to the relatively small number of RCTs. Therefore, large additional studies with variations in the amount of prescribed protein are needed to clarify the optimal dose of the intervention. There was evidence of high statistical heterogeneity for the primary mortality outcome. However, the exclusion of an outlying study from the analysis reduced the heterogeneity without significant change in the main finding. Moreover, units of protein intake varied in different studies (g/kg/day, %E, or g/day). There was no evidence of small study effects using Egger’s test; however, given the relatively low number of included studies in each endpoint, publication bias is still possible as the test was likely to be underpowered. Finally, our findings might not be generalizable to children, ICU patients with borderline liver function, those who are severely obese, and those with refractory hypotension or overwhelming sepsis. Moreover, while protein remains the central macronutrient in critical care, additional nutritional and sensory-based strategies—such as folate supplementation for psychological recovery, combined macronutrient intake with physical activity and mental engagement, or even olfactory stimulation through aromatherapy—may offer complementary benefits that are not fully reflected in traditional ICU outcomes.^69-71^

## Conclusion and future research

 The present systematic review and dose-response meta-analysis showed no meaningful effect of higher versus lower protein intake on mortality, infectious complications, mechanical ventilation duration, ICU, and hospital length of stay. Nonetheless, higher protein delivery, in comparison with lower protein delivery, significantly attenuated muscle loss. Future research should investigate the effect of higher doses of protein delivery to provide a more comprehensive evaluation of the dose-depending effects of protein intake on ICU outcomes as well as to examine whether the effect of high protein intake varies in different ICU patients.

## Data Availability Statement

 The data generated or analyzed during the current study are not publicly available but are available from the corresponding author upon reasonable request.

## Competing Interests

 There was no conflict of interest.

## Ethical Approval

 Not required.

## Supplementary Files

Supplementary file 1 contains Tables S1-S4 and Figures S1-S4.
