# Supplementary material for: Protein intake and clinical outcomes in critically ill patients: A dose–response and pairwise meta-analysis of randomized controlled trials
Source: Health Promot Perspect. 2025 Jul 15;15(2):98–109. doi: 10.34172/hpp.025.43894 (PMC12450574; doi:10.34172/hpp.025.43894)
Supplement: Supplementary file 1 — contains Tables S1-S4 and Figures S1-S4. [file hpp-15-98-s001.pdf]

Badpeyma et al, **Health Promotion Perspectives.** 2025;15(2):98-S1
doi: 10.34172/hpp.025.43894
https://hpp.tbzmed.ac.ir

Supporting file 1

Table S1: The terms used to search relevant articles on the association between higher versus lower protein delivery on clinical outcome.

Database	Step	Terms	Results
PubMed	1	"Critical Care"[MeSH Terms] OR "Critical Care"[All Fields] OR "Intensive Care"[All Fields] OR "Intensive Care Units"[MeSH Terms] OR "Intensive Care Unit"[All Fields] OR "ICU"[All Fields] OR "Critical Illness"[MeSH Terms] OR "Critical Illness"[All Fields] OR "Critical Illnesses"[All Fields] OR "Critically Ill"[All Fields] OR "Burn Units"[MeSH Terms] OR "Burn Units"[All Fields] OR "Burns"[MeSH Terms] OR "Burn"[All Fields] OR "Coronary Care Units"[MeSH Terms] OR "Coronary Care Unit"[All Fields] OR "Coronary Care"[All Fields] OR "Respiratory Care Units"[MeSH Terms] OR "Respiratory Care Unit"[All Fields] OR "Respiratory Care"[All Fields] OR "Sepsis"[MeSH Terms] OR "Sepsis"[All Fields] OR "Bloodstream Infection"[All Fields] OR "Septicemia"[All Fields] OR "Pyemia"[All Fields] OR "Systemic Inflammatory Response Syndrome"[MeSH Terms] OR "Systemic Inflammatory Response Syndrome"[All Fields] OR "Multiple Organ Failure"[MeSH Terms] OR "Multiple Organ Failure"[All Fields] OR "Multiple Organ Dysfunction Syndrome"[All Fields] OR "MODS"[All Fields] OR "Respiratory Distress Syndrome"[MeSH Terms] OR "Respiratory Distress Syndrome"[All Fields] OR "ARDS"[All Fields] OR "Severe Acute Respiratory Syndrome"[MeSH Terms] OR "Severe Acute Respiratory Syndrome"[All Fields] OR "SARS"[All Fields] OR "Acute Lung Injury"[MeSH Terms] OR "Acute Lung Injury"[All Fields] OR "Multiple Trauma"[MeSH Terms] OR "Multiple Trauma"[All Fields] OR "Multiple Injury"[All Fields] OR "Polytrauma"[All Fields] OR "Multiple Wound"[All Fields] OR "Abdominal Injuries"[MeSH Terms] OR "Abdominal Injury"[All Fields] OR "Pancreatitis"[MeSH Terms] OR "Pancreatitis"[All Fields] OR "Brain Injuries"[MeSH Terms] OR "Brain Injury"[All Fields] OR "Traumatic Brain Hemorrhage"[All Fields] OR "Traumatic brain"[All Fields] OR "Diffuse Cerebral Injury"[All Fields] OR "Craniocerebral Trauma"[MeSH Terms] OR "Craniocerebral Trauma"[All Fields] OR "Head Trauma"[All Fields] OR "Head Injury"[All Fields]	
	2	"Nutrition Therapy"[MeSH Terms] OR "Nutrition Therapy"[All Fields] OR "Nutritional Support"[MeSH Terms] OR "Nutritional Support"[All Fields] OR "Artificial Feeding"[All Fields] OR "Enteral Nutrition"[MeSH Terms] OR "Enteral Nutrition"[All Fields] OR "Enteral Feeding"[All Fields] OR "Tube Feeding"[All Fields] OR "Parenteral Nutrition"[MeSH Terms] OR "Parenteral	

		Nutrition"[All Fields] OR "Parenteral Feeding"[All Fields] OR "Intravenous Feeding"[All Fields] OR "intubation, gastrointestinal"[MeSH Terms] OR "Gastrointestinal Intubation"[All Fields] OR "Nasogastric Intubation"[All Fields] OR "Parenteral Nutrition Solutions"[MeSH Terms] OR "Intravenous Feeding Solutions"[All Fields] OR "Dietary Proteins"[MeSH Terms] OR "Dietary Protein"[All Fields] OR "Protein Intake"[All Fields] OR "Protein Consumption"[All Fields] OR "Dietary Amino Acid"[All Fields] OR "Amino Acid Intake"[All Fields] OR "Protein"[Title] OR "Amino Acid"[Title]	
	3	"Randomized Controlled Trial"[Publication Type] OR "Controlled Clinical Trial"[Publication Type] OR "Clinical Trial"[Publication Type] OR "Clinical Trials as Topic"[MeSH Terms] OR "Random Allocation"[MeSH Terms] OR "Randomization"[All Fields] OR "Randomized"[All Fields] OR "Placebo"[All Fields] OR "Randomly"[All Fields] OR "Trial"[All Fields] OR "RCT"[All Fields] OR "Placebos"[MeSH Terms])) AND (2021:2023[pdat]	
	4	#1 AND #2 AND #3	769
Scopus	1	TITLE-ABS-KEY ("Randomized Controlled Trial") OR TITLE-ABS-KEY ("Controlled Clinical Trial") OR TITLE-ABS-KEY ("Clinical Trial") OR TITLE-ABS-KEY ("Clinical Trials as Topic") OR TITLE-ABS-KEY ("Random Allocation") OR TITLE-ABS-KEY ("Randomization") OR TITLE-ABS-KEY ("Randomized") OR TITLE-ABS-KEY ("Placebo") OR TITLE-ABS-KEY (randomly) OR TITLE-ABS-KEY (trial) OR TITLE-ABS-KEY (rct) OR TITLE-ABS-KEY (placebos)	
	2	TITLE-ABS-KEY ("Nutrition Therapy") OR TITLE-ABS-KEY ("Nutritional Support") OR TITLE-ABS-KEY ("Artificial Feeding") OR TITLE-ABS-KEY ("Enteral Nutrition") OR TITLE-ABS-KEY ("Enteral Feeding") OR TITLE-ABS-KEY ("Tube Feeding") OR TITLE-ABS-KEY ("Parenteral Nutrition") OR TITLE-ABS-KEY ("Parenteral Feeding") OR TITLE-ABS-KEY ("Intravenous Feeding") OR TITLE-ABS-KEY ("Gastrointestinal Intubation") OR TITLE-ABS-KEY ("Nasogastric Intubation") OR TITLE-ABS-KEY ("Parenteral Nutrition Solutions") OR TITLE-ABS-KEY ("Intravenous Feeding Solutions") OR TITLE-ABS-KEY ("Dietary Proteins") OR TITLE-ABS-KEY ("Protein Intake") OR TITLE-ABS-KEY ("Protein Consumption") OR TITLE-ABS-KEY ("Dietary Amino Acid") OR TITLE-ABS-KEY (protein) OR TITLE-ABS-KEY ("Amino Acid")	

	3	TITLE-ABS-KEY ("Critical Care") OR TITLE-ABS-KEY ("Intensive Care") OR TITLE-ABS-KEY ("Intensive Care Unit") OR TITLE-ABS-KEY (icu) OR TITLE-ABS-KEY ("Critical Illness") OR TITLE-ABS-KEY ("Critically Ill") OR TITLE-ABS-KEY ("Burn Units") OR TITLE-ABS-KEY (burn) OR TITLE-ABS-KEY ("Coronary Care Units") OR TITLE-ABS-KEY ("Coronary Care") OR TITLE-ABS-KEY ("Respiratory Care Unit") OR TITLE-ABS-KEY ("Respiratory Care") OR TITLE-ABS-KEY (sepsis) OR TITLE-ABS-KEY ("Bloodstream Infection") OR TITLE-ABS-KEY (septicemia) OR TITLE-ABS-KEY (pyemia) OR TITLE-ABS-KEY ("Systemic Inflammatory Response Syndrome") OR TITLE-ABS-KEY ("Multiple Organ Failure") OR TITLE-ABS-KEY ("Multiple Organ Dysfunction Syndrome") OR TITLE-ABS-KEY (mods) OR TITLE-ABS-KEY ("Respiratory Distress Syndrome") OR TITLE-ABS-KEY (ards) OR TITLE-ABS-KEY ("Severe Acute Respiratory Syndrome") OR TITLE-ABS-KEY (sars) OR TITLE-ABS-KEY ("Acute Lung Injury") OR TITLE-ABS-KEY ("Multiple Trauma") OR TITLE-ABS-KEY ("Multiple Injury") OR TITLE-ABS-KEY (polytrauma) OR TITLE-ABS-KEY ("Multiple Wound") OR TITLE-ABS-KEY ("Abdominal Injuries") OR TITLE-ABS-KEY ("Abdominal Injury") OR TITLE-ABS-KEY (pancreatitis) OR TITLE-ABS-KEY ("Brain Injuries") OR TITLE-ABS-KEY ("Brain Injury") OR TITLE-ABS-KEY ("Traumatic Brain Hemorrhage") OR TITLE-ABS-KEY ("Traumatic brain") OR TITLE-ABS-KEY ("Diffuse Cerebral Injury") OR TITLE-ABS-KEY ("Craniocerebral Trauma") OR TITLE-ABS-KEY ("Head Trauma") OR TITLE-ABS-KEY ("Head Injury")))	
	4	#1 AND #2 AND #3	**5923**
Web of Science	1	TS=("Critical Care" OR "Intensive Care" OR "Intensive Care Unit" OR icu OR "Critical Illness" OR "Critically Ill" OR "Burn Units" OR burn OR "Coronary Care Units" OR "Coronary Care" OR "Respiratory Care Unit" OR "Respiratory Care" OR sepsis OR "Bloodstream Infection" OR septicemia OR premia OR "Systemic Inflammatory Response Syndrome" OR "Multiple Organ Failure" OR "Multiple Organ Dysfunction Syndrome" OR mods OR "Respiratory Distress Syndrome" OR ards OR "Severe Acute Respiratory Syndrome" OR sars OR "Acute Lung Injury" OR "Multiple Trauma" OR "Multiple Injury" OR polytrauma OR "Multiple Wound" OR "Abdominal Injuries" OR "Abdominal Injury" OR pancreatitis OR "Brain Injuries" OR "Brain Injury" OR "Traumatic Brain Hemorrhage" OR "Traumatic brain" OR "Diffuse Cerebral Injury" OR "Craniocerebral Trauma" OR "Head Trauma" OR "Head Injury")	

	2	TS=("Nutrition Therapy" OR "Nutritional Support" OR "Artificial Feeding" OR "Enteral Nutrition" OR "Enteral Feeding" OR "Tube Feeding" OR "Parenteral Nutrition" OR "Parenteral Feeding" OR "Intravenous Feeding" OR "Gastrointestinal Intubation" OR "Nasogastric Intubation" OR "Parenteral Nutrition Solutions" OR "Intravenous Feeding Solutions" OR "Dietary Proteins" OR "Protein Intake" OR "Protein Consumption" OR "Dietary Amino Acid" OR protein OR "Amino Acid")	
	3	TS=("Randomized Controlled Trial" OR "Controlled Clinical Trial" OR "Clinical Trial" OR "Clinical Trials as Topic" OR "Random Allocation" OR "Randomization" OR "Randomized" OR "Placebo" OR randomly OR trial OR rct OR placebos)	
	4	#1 AND #2 AND #3	**2119**
Total			8811

Table S2: Characteristics of included studies on the effects of higher versus lower protein delivery in critically ill patients.

Author, year	Location	Population	Sample size	Route nutrition therapy		Intervention			Measured outcomes
				EN	PN	Protein		Energy	
						Treatment group	Control group		
Clifton 1985 [36]	USA	Severe head injury	M/F: 20	EN only		22% calories from protein (Traumacal)	14% calories from protein (Magnacal)	IC	Overall mortality, Infectious complications
Mesejo 2003 [37]	Spain	EN ≥ 5 days, APACHE II 10–25, BMI ≥ 30, no kidney/liver failure	M/F: 50	EN only		22% calories from protein (Protein: 33g) (Isosource Protein)	20% calories from protein (Protein: 30g) (Novasource Diabet Plus)	Harris-benedict equation*1.2	Overall mortality, Infectious complications, Duration of MV, ICU LOS
Zhou 2006 [38]	China	Severe stroke with GCS < 12	M/F: 51	EN only		NPC: N = 100:1 20% calories from protein	NPC: N=130:1 16% calories from protein (Nutrition Fibre)	104.5 kJ · kg -1 · d -1 (25 kcal/kg)	Overall mortality

						(Fresubin 750 MCT)			
Singer 2007 [8]	Israel	MV with non-oliguric acute renal failure and required PN	M/F: 14		PN only	75 g of amino acids (Aminoplasmal 10%, B Braun, Germany)	150 g of amino acids (Aminoplasmal 10%, B Braun, Germany)	2000 non-protein kcal/ day (dextrose and Intralipid, Fresenius-Kabi, Germany)	Overall mortality
Rugeles 2013 [39]	Columbia	Medical adult ICU patients , EN ≥ 96 h	M/F: 80	EN only	Exclude patients that need PN	hypocaloric hyperproteic (15 kcal/kg, >1.5 g/kg/d) x 7 days	Standard (25 kcal/kg, 20% calories from protein	Harris-benedict equation	Duration of MV, ICU LOS
Doig 2015 [40]	Australia	Mixed, stay≥ 2d	M/F: 474		Decide by the attending physician	100 g/L (100 g/d) of L-amino acids up to 2.0 g/kg/d (Synthamin 17 Electrolyte Free, Baxter Healthcare, Australia)	Standard care	If BMI> 25, use IBW at BMI 23 / Decide by the attendi ng clinicia ns	Overall mortality
Ferrie 2015 [41]	Australia	Mixed, ≥ 3d on PN	M/F: 120		PN only	1.2 g/kg aa Olimel N9(Baxter,Lessines , Belgium)	0.8 g/kg aa Oli-Clinomel N7(Baxter, Lessines, Belgium)	25 kcal/kg/d	Overall mortality, Duration of MV, ICU LOS, Hospital LOS, Muscle outcome
Jakob 2017 [42]	Switzerlan d	Mixed, EN ≥ 3d, stay ≥ 5d	M/F: 90	EN first	PN is only allowed if intolerant to EN	94 g protein/liter, 25% calories from protein (Peptamen® AF)	61 g protein/liter; 16% calories from protein (Isosource Energy)	25 kcal/kg/d and adjusted by IC	Infectious complications, Duration of MV, ICU LOS, Hospital LOS
Fetterplac e 2018 [43]	Australia	Mixed, MV within 48 h and remained ≥ 72 h	M/F: 60	EN first	PN is allowed at the	1.5 g/kg/d from high protein EN (Nutrison Protein Plus)	1.0 g/kg/d from standard EN (Nutrison)	25 kcal/kg/d	Overall mortality, Duration of MV, ICU LOS, Hospital LOS, Muscle outcome

van Zanten 2018 [44]	Netherlands	Mixed, MV, BMI ≥ 25, EN ≤ 48 h- > 5d	M/F: 44	EN first	SPN is allowed if necessary	Very High Protein Formula (VHPF): 10g/100ml Protein (32%)	Standard High Protein Formula (SHPF): 5 g g/100ml Protein (20%) (Nutrison Protein Plus; Nutricia, Zoetermeer, The Netherlands)	25 kcal/kg IBW/d		Overall mortality, Duration of MV, ICU LOS, Hospital LOS
Vega-Alava 2018 [45]	Philippines	MV, EN	M/F: 40	EN only		10 g protein per serving and 16% of calories from protein) + 100% whey protein supplement (6 g protein per serving), 3 servings, every 8 hour (Peptamen + Beneprotein)	whey protein (10 g protein per serving and 16% of calories from protein) only Peptamen	25-30 kcal/kg IBW/d * stress factor		Infectious complications
Azevedo 2019 [46]	Brazil	Mixed, MV, Stay > 2d	M/F: 120	EN first	SPN is allowed after	2.0-2.2 g/kg/d	1.-1.5 g/kg/d	IC	25 kcal/kg /d	Overall mortality, Duration of MV, ICU LOS
Danielis 2019 [7]	Italy	Mixed, MV within 12 h, BMI 18.5 to 30, no acute/chronic renal or hepatic failure	M/F: 40	EN first	SPN is allowed to make	1.8 g/kg/d EN & PN:16% of calories from protein	Standard Diet EN: 21% of calories from protein PN: 22% of calories from protein	20-25 kcal/kg/d		Overall mortality, Duration of MV, ICU LOS
Badjatia 2020 [47]	USA	SAH, Stay > 7d, BMI 15 to 40	M/F: 25	EN or oral intake		1.75 g/kg/d (≥9g leucine/d)	1.2-1.4 g/kg/d	Mifflin St-Jeor (non-intubated)		Infectious complications, ICU LOS, Muscle outcome
Bukhari 2020^ [48]	Indonesia	ICU patients not contraindicated or intolerant to EN	M/F: 33	EN only		22.4% calories from protein (Peptisol)	16.2% calories from protein (Peptamen)	25-30 kcal/kg/d		Overall mortality, ICU LOS, Hospital LOS
Chapple 2020 [49]	Australia	Mixed, MV, EN > 2d	M/F: 116	EN first	SPN is allowed if deemed	EN formula with 100 g protein/liter; 32% of calories	EN formula with 63 g protein/liter; 20% of calories from	1260 kcal/liter	1250 kcal/liter	Overall mortality, ICU LOS, Hospital LOS

					necessary by the treating physician	from protein (Nutrison Protein Intense)	protein (Nutrison Protein Plus)		
Nakamura 2020 [50]	Japan	Mixed, No lower limb injury, No die or discharge < 10 d	M/F: 117	EN first	SPN is allowed to reach	1.8 g/kg/d	0.9 g/kg/d	20 kcal/kg/d (initial target), up to 30 kcal/kg/d if malnutrition (defined as BMI<18.5, recent weight loss>10%, or physician's decision)	Overall mortality, Duration of MV, Hospital LOS, Muscle outcome
Carteron 2021 [51]	France	Brain injured (GCS <8), expected MV > 48 h	M/F: 195	EN only		EN formula with 9.4 g of hydrolyzed protein per 100 ml	EN formula with 7.5 g of protein per 100 ml	Lorentz's Formula	Overall mortality, Infectious complications, Duration of MV, ICU LOS
Dresen 2021 [52]	Germany	Surgical, MV, after stay ≥ 10d, expected stay ≥ 30d	M/F: 42	EN first	If nutrition targets were not achieved within 24 h, initiate SPN	1.8 g protein/kg body weight [B.W.]/d	1.2 g protein/kg BW/d	Repeated IC or Harris-Benedict.	Overall mortality, Infectious complications, Duration of MV, ICU LOS, Muscle outcome
Azevedo, 2021 [53]	Brazil	Mixed, MV, stay > 3d	M/F: 181	EN first	PN is allowed after 7 days if the caloric goal not achieved	1.48 g/kg/d	1.19 g/kg/d	IC	Overall mortality, Duration of MV, ICU LOS
Kagan, 2021^^ [54]	Israel	Mixed, MV within 48 h and expected to	M/F: 40	EN	PN	25% of calories from protein (Groups 2= EN (Jevity®, Abbott))	16.7% of calories from protein protein-enriched EN (very high-protein	IC	Overall mortality, Duration of MV, ICU LOS, Hospital LOS

		remain a minimum of 7 d					formula Promote®, Abbott)		
Uyar, 2022 [1]	Turkey	Mixed	M/F: 54	EN only		2 g/kg/d	1.2 g/kg/d	25 kcal/kg/d	Overall mortality
Heyland, 2023 [20]	Multicenter**	Mixed*	M/F: 1301	EN first	EN combination with PN, intravenous amino acids	≥2·2 g/kg/d	≤1·2 g/kg/d	If BMI>30, use IBW at BMI 25	Overall mortality, Duration of MV, ICU LOS, Hospital LOS

^ This study has 3 groups: the control (*n* = 22), the high-protein polymeric (*n* = 19), and the oligomeric group (*n* = 14); the control group was excluded from the analysis.
^^ This study has 3 groups: control (*n* = 22), CE with EN (*n* = 21) and CE with protein-enriched EN (*n* = 19); the control group was excluded from the analysis.
Abbreviations: EN: enteral nutrition, *PN:* parenteral nutrition, aa: amino acid, *d:* day(s), *h* hour, *ICU:* intensive care unit, *LOS:* length of stay, *SPN:* supplemental parenteral nutrition, *MV:* mechanical ventilation, *BMI:* body mass index, *IC:* Indirect Calorimetry, *M/F*: Male/Female, *MCT*: medium chain triglycerides.
* One or more nutritional risk factors: (1) low (≤25 kg/m²) or high (≥35 kg/m²) BMI; (2) moderate to severe malnutrition, as defined by local assessments; frailty, as defined by a Clinical Frailty Scale of 5 or more from proxy; (4) sarcopenia, as defined by a SARC-F score of 4 or more from proxy; and (5) from point of screening, projected duration of mechanical ventilation of more than 4 days.
** Undertaken in 85 intensive care units (ICUs) across 16 countries, including Argentina, Australia, Brazil, Canada, China, Greece, India, Iran, Japan, Malaysia, Mexico, Panama, Puerto Rico, Saudi Arabia, the U.K., and the USA.

Table S3: Results of risk of bias assessment for randomized clinical trials included in the current meta-analysis on the effects of high-protein nutritional support on clinical outcomes in critically ill patients [1]

	Random Sequence Generation	Allocation concealment	Selective reporting	Other sources of bias	Blinding (participants and personnel)	Blinding (outcome assessment)	Incomplete outcome data	Overall
Clifton et al. 1985 [36]	U	U	L	L	H	L	L	U
Mesejo et al. 2003 [37]	L	L	L	L	H	L	L	U
Zhou et al. 2006 [38]	H	U	L	L	H	L	L	H

Singer et al. 2007 [8]	U	U	L	L	H	L	L	U
Rugeles et al. 2013 [39]	L	L	L	L	L	L	H	U
Doig et al. 2015 [40]	L	L	L	L	H	L	H	H
Ferrie et al. 2015 [41]	L	L	L	L	L	L	L	L
Jakob et al. 2017 [42]	L	L	L	L	L	L	L	L
Fetterplace et al. 2018 [43]	L	L	L	L	H	L	L	U
Van Zanten et al. 2018 [44]	L	L	L	L	L	L	L	L
Vega-Alava et al. 2018 [45]	U	L	L	L	H	L	L	U
Azevedo et al. 2019 [46]	L	L	L	L	H	L	H	H
Danielis et al. 2019 [7]	L	U	L	L	H	L	L	U
Badjatia et al. 2020 [47]	L	U	L	L	H	L	L	U
Bukhari et al. 2020 [48]	U	U	L	L	H	L	H	H
Chapple et al. 2020 [49]	L	L	L	L	H	L	H	H
Nakamura et al. 2020 [50]	L	U	L	L	H	L	H	H
Carteron et al. 2021 [51]	L	L	L	L	H	L	H	H
Dresen et al. 2021 [52]	L	U	L	L	H	L	H	H
Azevedo et al. 2021[53]	L	L	L	L	H	L	H	H
Kagan et al. 2021 [54]	L	L	L	L	H	L	L	U
Uyar et al. 2022 [1]	L	U	L	L	H	L	L	U
Heyland et al. 2023 [20]	L	L	L	L	H	L	L	U

[1] Each study was assessed for risk of bias using the Cochrane Risk of Bias Assessment tool. Assessment domains included random sequence generation, allocation concealment, reporting bias, performance bias, detection bias, attrition bias, and other sources of bias. Each domain was scored as "high risk" if it contained methodological flaws that may have

affected the results, "low risk" if the flaw was deemed inconsequential, and "unclear risk" if the information was insufficient to determine. Low risk of bias: ≤1 items was unknown, and none were high; Some concern: ≤2 items were unclear or one item was high; High risk of bias: ≥2 items were high.
Abbreviations: U; unclear risk of bias, L; low risk of bias, H; high risk of bias.

Table S4: Stratified analyses on the effects of High-Protein on clinical outcomes in critically ill patients in adults aged >18 years.

	n[2]	Pooled RR (95% CI)[3]	I^2 (%)[4]	P[5]
	**Binary outcomes**			
		**Overall mortality**		
Location				
Western	13	0.77 (0.55 to 1.08)	74.8	<0.001
Non-Western	6	1.00 (0.65 to 1.53)	0	0.91
Feeding				
EN	16	0.79 (0.58 to 1.09)	68.9	<0.001
PN	3	0.94 (0.67 to 1.33)	0	0.56
		**Infectious complications**		
Location				
Western	6	1.06 (0.89 to 1.26)	0	0.80
Non-Western	1	0.09 (0.01 to 1.54)	-	-
Feeding				
EN	7	1.05 (0.88 to 1.25)	0	0.44
PN	0	-	-	-
**Continues outcomes**				
	n***	**MD (95% CI)†**	**I^2 (%)‡**	**P§**
		**Duration of MV[1]**		
Location				
Western	12	-0.07 (-0.69 to 0.54)	8.3	0.36
Non-Western	2	-0.88 (-1.96 to 0.21)	0	0.83
Feeding				
EN	13	-0.27 (-0.78 to 0.24)	2.6	0.42
PN	1	2.20 (-1.78 to 6.18)	-	-
		**ICU LOS[1]**		
Location				
Western	13	-0.21 (-1.03 to 0.61)	0	0.87
Non-Western	4	-0.95 (-2.39 to 0.48)	0	0.55
Feeding				
EN	16	-0.48 (-1.20 to 0.24)	0	0.93
PN	1	2.58 (-1.69 to 6.85)	-	-
		**Hospital LOS[1]**		
Location				
Western	7	0.98 (-0.90 to 2.86)	0	0.60
Non-Western	3	-2.11 (-11.55 to 7.34)	43.9	0.16
Feeding				

EN	9	0.68 (-1.52 to 2.89)	9.2	0.35
PN	1	4.05 (-9.11 to 17.21)	-	-
		**Muscle atrophy**		
Location				
Western	4	-0.57 (-1.34 to -0.20)	80.7	0.001
Non-Western	2	-0.37 (-0.74 to -0.01)	24.4	0.25
Feeding				
EN	5	-0.60 (-0.95 to -0.24)	43.3	0.13
PN	1	0.38 (-0.15 to 0.90)	-	-

[1] Abbreviations: MV: Mechanical Ventilation, LOS: Length of stay, EN: Enteral Nutrition, PN: Parenteral Nutrition, CI: confidence interval, MD, mean difference, RR: risk ratio.
[2] Number of studies
[3] Obtained from the random-effects model
[4] Inconsistency- the percentage of variation across studies due to heterogeneity
[5] Obtained from Q test

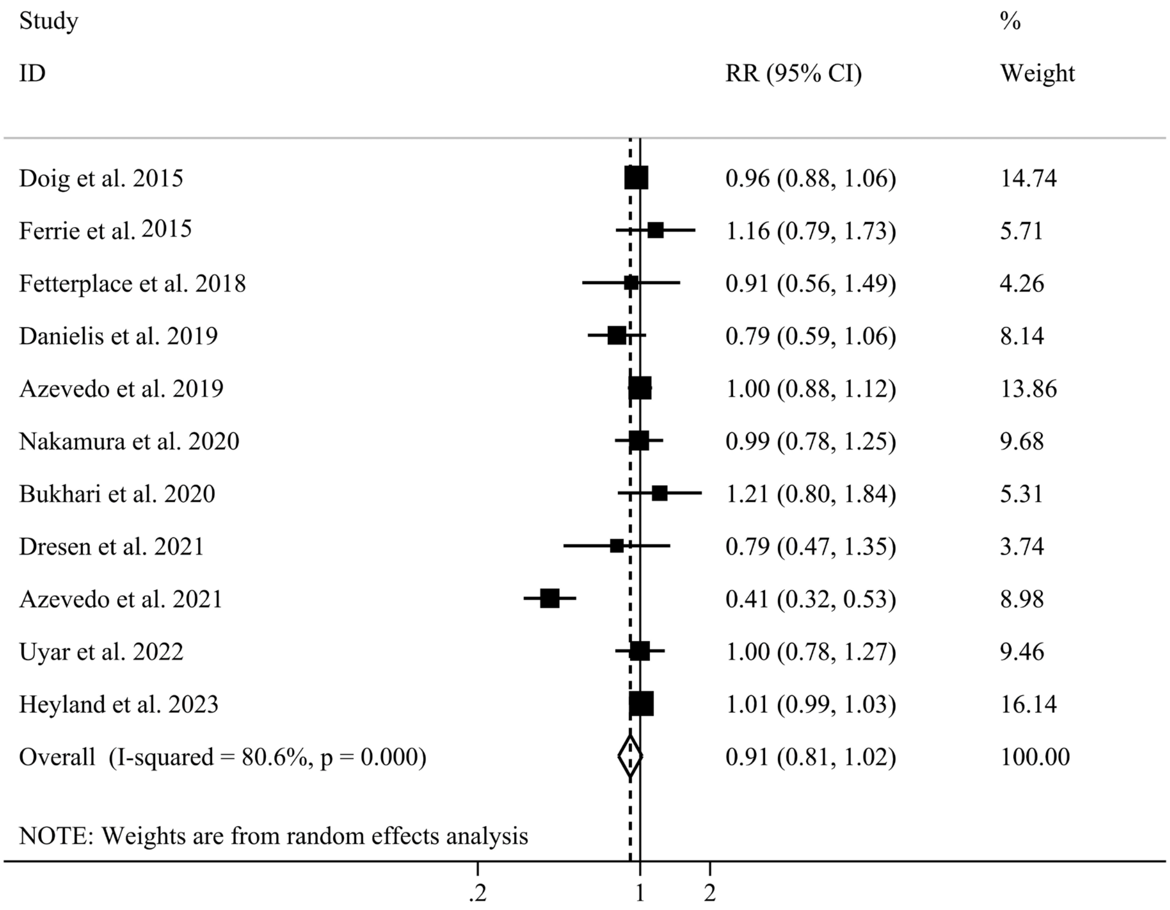

Figure S1. Forest plot for the overall mortality based on 0.2 g increase in protein intake in ICU patients. Horizontal lines represent 95% CIs Diamonds represent the pooled estimates from the random-effects analysis; LOS, Length of stay; ICU, Intensive care unit; WMD, weighted mean difference; CI: confidence interval.

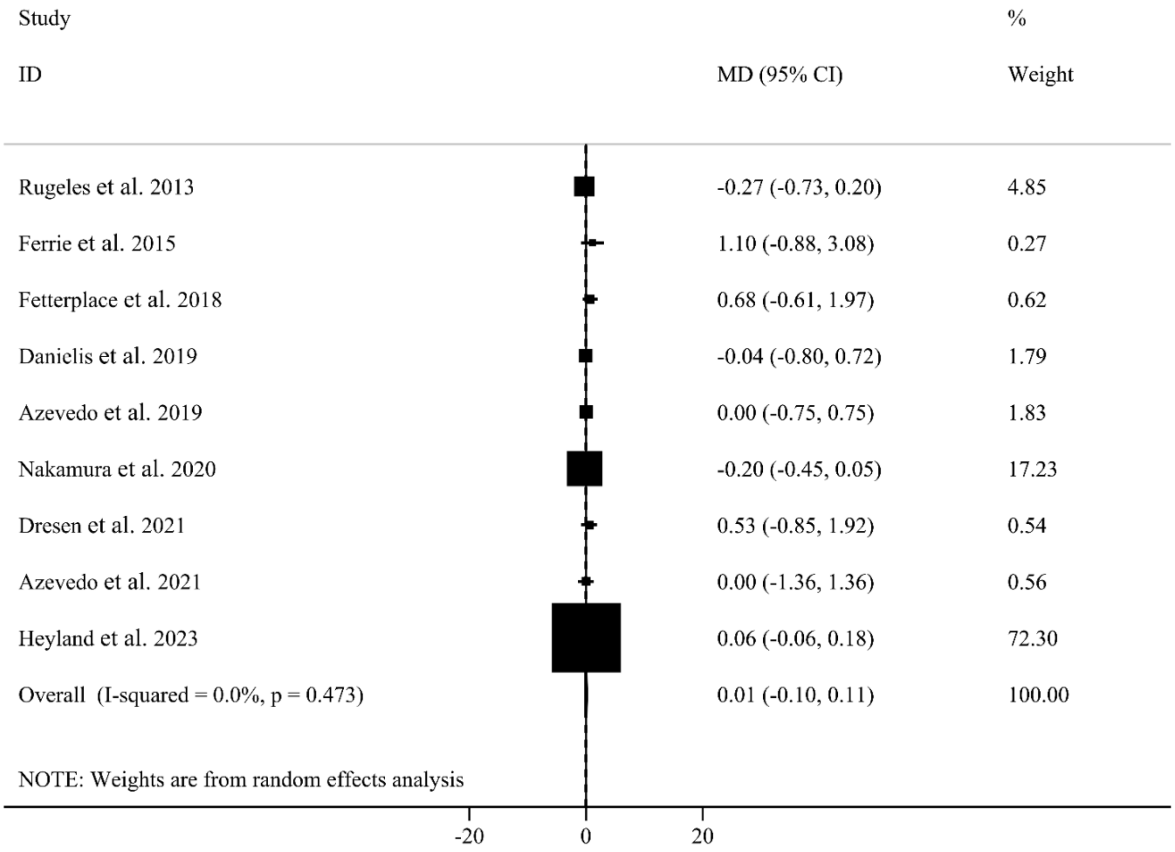

Figure S2. Forest plot for the MV Ventilation based on 0.2 g increase in protein intake in ICU patients. Horizontal lines represent 95% CIs Diamonds represent the pooled estimates from the random-effects analysis. ICU, Intensive care unit; MV: Mechanical Ventilation; WMD, weighted mean difference; CI: confidence interval.

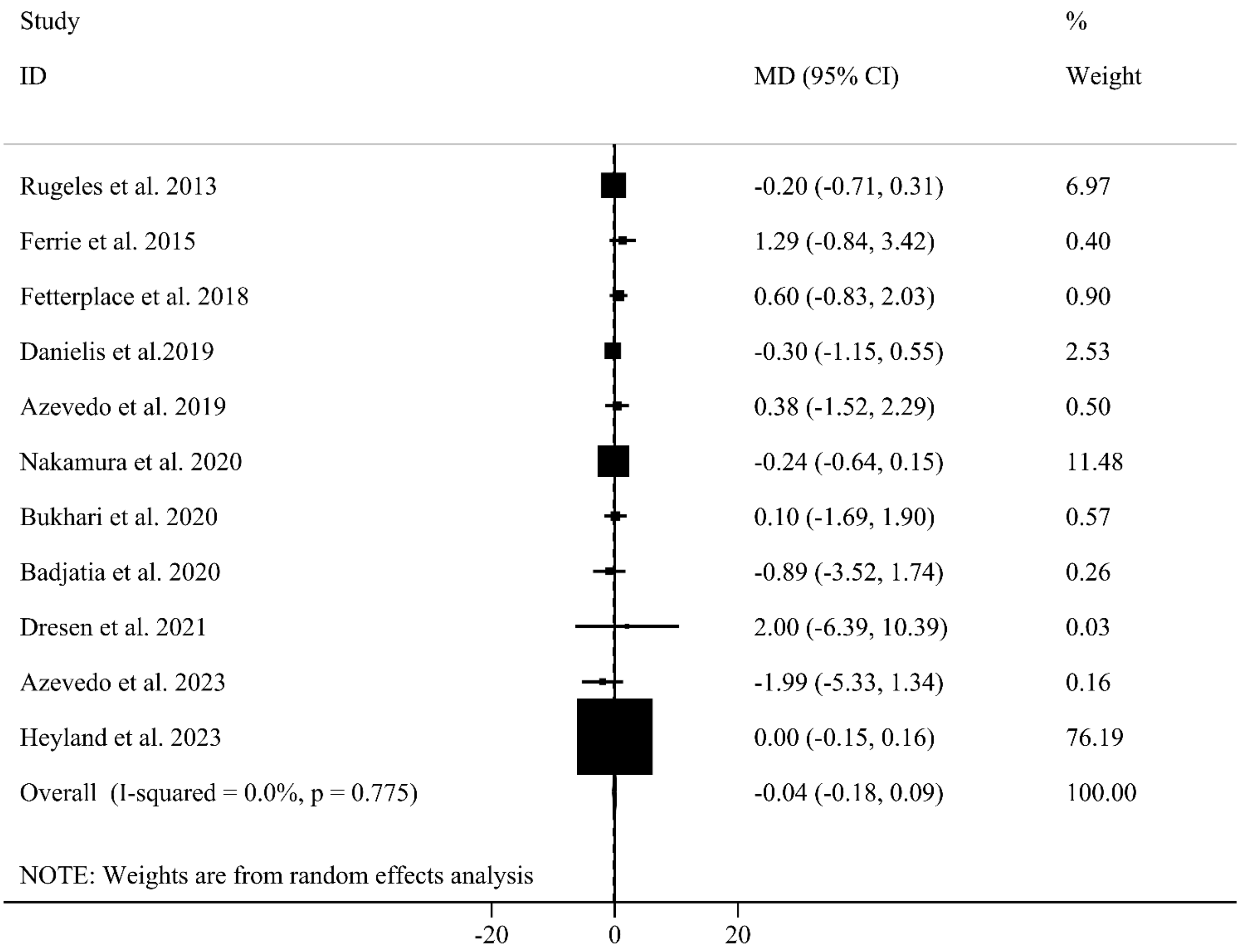

Figure S3. Forest plot for the ICU LOS based on 0.2 g increase in protein intake in ICU patients. Horizontal lines represent 95% CIs Diamonds represent the pooled estimates from the random-effects analysis. ICU, Intensive care unit; LOS, Length of stay; WMD, weighted mean difference; CI: confidence interval.

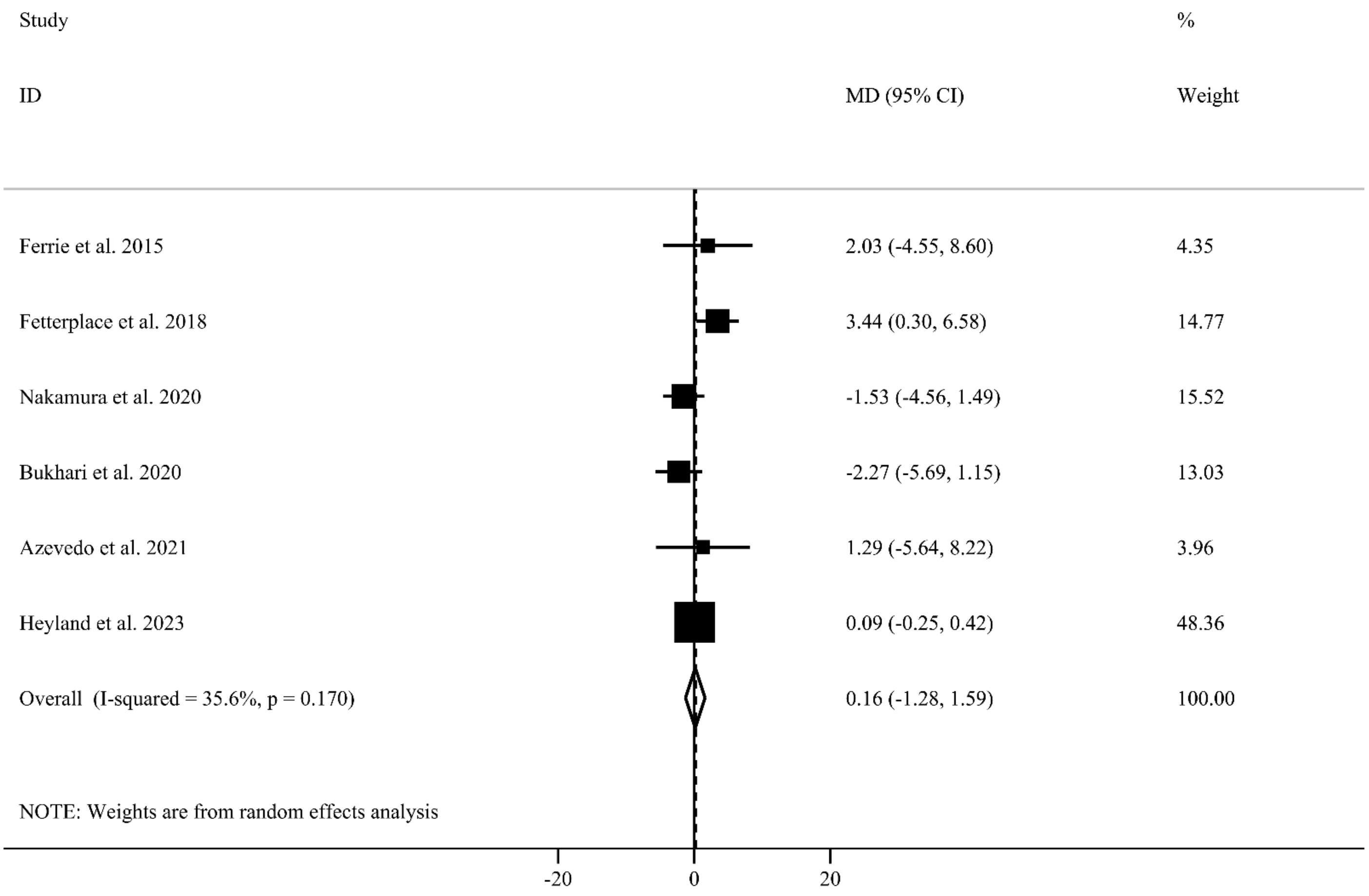

Figure S4. Forest plot for the hospital LOS based on 0.2 g increase in protein intake in ICU patients. Horizontal lines represent 95% CIs Diamonds represent the pooled estimates from the random-effects analysis; LOS, Length of stay; ICU, Intensive care unit; WMD, weighted mean difference; CI: confidence interval.
